# Functional Conversion of Acetyl-Coenzyme a Synthase to a Nickel Superoxide Dismutase via Rational Design of Coordination Microenvironment for the Ni_d_-Site

**DOI:** 10.3390/ijms23052652

**Published:** 2022-02-28

**Authors:** Yaozhu Wei, Yajun Zhou, Hong Yuan, Yi Liu, Ying-Wu Lin, Jihu Su, Xiangshi Tan

**Affiliations:** 1 Department of Chemistry, Fudan University, Shanghai 200433, China; 12110220023@fudan.edu.cn (Y.W.); 15110220061@fudan.edu.cn (Y.Z.); yuanhong119@163.com (H.Y.); yiliu0825@hubu.edu.cn (Y.L.); 2 School of Chemistry and Chemical Engineering, University of South China, Hengyang 421001, China; ywlin@usc.edu.cn; 3 Department of Modern Physics, University of Science and Technology of China, Hefei 230026, China; sujihu@ustc.edu.cn

**Keywords:** metalloenzyme, metalloprotein, protein molecular design, acetyl-coenzyme A synthase, Ni-SOD

## Abstract

The Ni_d_ site coordination microenvironment of a truncated acetyl-coenzyme A synthase has been designed systematically for functional conversion to a Ni-SOD-like enzyme. To this end, the first strategy is to introduce an axial histidine ligand, using mutations F598H, S594H and S594H-GP individually. The resulting three mutants obtained Ni-SOD-like activity successfully, although the catalytic activity was about 10-fold lower than in native Ni-SOD. The second strategy is to mimic the H-bond network in the second sphere coordination microenvironment of the native Ni-SOD. Two mutations based on F598H (EFG-F598H and YGP-F598H) were designed. The successful EFG-F598H exhibited ~3-fold Ni-SOD-like activity of F598H. These designed Ni-SOD-like metalloproteins were characterized by UV/Vis, EPR and Cyclic voltammetry while F598H was also characterized by X-ray protein crystallography. The pH titrations were performed to reveal the source of the two protons required for forming H_2_O_2_ in the SOD catalytic reaction. Based on all of the results, a proposed catalytic mechanism for the Ni-SOD-like metalloproteins is presented.

## 1. Introduction

The functional diversity of metalloenzymes/metalloproteins is imparted mainly by the incorporated metal centers along with the versatility of the coordination microenvironment, including different oxidation states, metal ion coordination geometry and the hydrogen bonding network in coordination environment [1,2]. Rational design of structural and functional metalloenzymes would greatly advance the field of protein engineering and provide a rigorous test of our knowledge of enzymes [3]. Successful functional conversion of metalloenzymes by molecular design is a challenge to reveal critical structural and mechanistic features that may be concealed in native metalloenzymes [4,5,6,7,8,9,10]. Eventually, these features would provide new insights into the functional evolution of metalloenzymes [1,2,3].

Acetyl-coenzyme A synthase (ACS) catalyzes the synthesis of acetyl-coenzyme A by condensing CO, coenzyme A and a methyl group from CH_3_-CoFeSP protein at the A-cluster, which consists of a [Fe_4_S_4_] Cubane bridged to a [Ni_p_Ni_d_] subcomponent via the thiolate of Cys509 [11,12,13]. The proximal nickel, called Ni_p_ site, coordinated to three cysteine thiolates and an unidentified exogenous ligand, is labile and can be removed by treatment with 1,10-phenanthroline (phen). The distal square-planar nickel, called Ni_d_ site, utilizes two deprotonated amides and two cysteine thiolates to form a square-planar nickel center [Ni_d_N_2_S_2_] connected to a Cys-Gly-Cys motif. Although the Ni_p_ site, as the catalytic metal for acetyl-CoA synthesis, has been well-studied, the function of the Ni_d_ site is not clear yet, and the Ni_d_ is believed to be a redox-inactive metal and remains in the Ni^2+^ state throughout the catalytic cycle [12].

Nickel superoxide dismutase (Ni-SOD) is also a nickel-containing protein, and its active site in reduced form resembles the [Ni^2+^N_2_S_2_] coordination environment of the Ni_d_ site of ACS, though they exhibit different properties and functions [14,15]. Ni-SOD is a metalloenzyme that disproportionately superoxides into O_2_ and H_2_O_2_, and its Ni center switches between Ni^2+^ and Ni^3+^ states during the catalytic cycle. In the reduced state, the Ni^2+^ adopts a square planar coordination geometry composed of two thiolates, a deprotonated amide nitrogen and the N-terminal amine nitrogen. Upon oxidation, the Ni^3+^ center is converted to square-pyramidal with His imidazole forming the fifth coordination bond [14,16].

**Figure 1:**
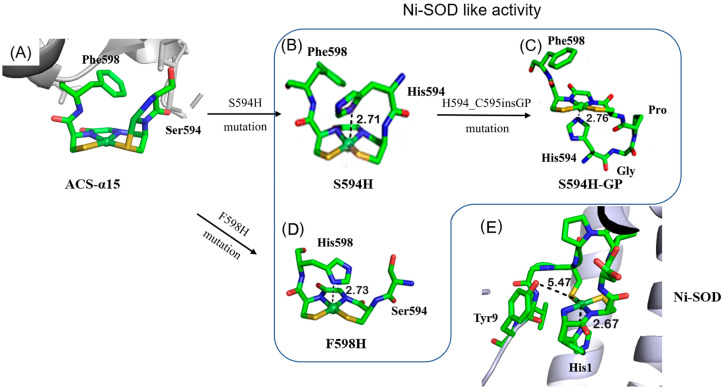
X-ray crystal structure of the truncated ACS protein ACS-α_15_ (PDB: 3S2X, from *Moorella thermoacetica*.) (**A**); modeling structure of the designed Ni-SOD-like metalloprotein S594H (**B**); S594H-GP (**C**); F598H (**D**); and X-ray crystal structure of Ni-SOD (PDB: 1T6U, from *Streptomyces coelicolor*.) (**E**).

**Figure 2:**
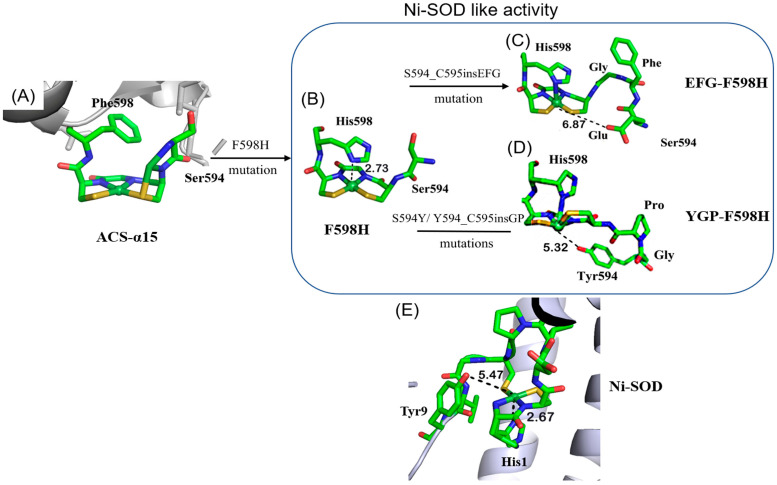
X-ray crystal structure of the truncated ACS protein ACS-α_15_ (PDB: 3S2X) (**A**); modeling structure of the designed Ni-SOD-like metalloprotein F598H (**B**); EFG-F598H (**C**); YGP-F598H (**D**); and X-ray crystal structure of Ni-SOD (PDB: 1T6U) (**E**).

Although the Ni_d_ site of ACS resembles the [NiN_2_S_2_] coordination environment in reduced Ni-SOD, it does not exhibit any detectable SOD activity [17]. In this work, we aim to convert the redox-inactive Ni_d_ site of a truncated ACS into a redox-active Ni-SOD-like metalloenzyme. A series of molecular designs have been carried out systematically, based on the redox-inactive Ni_d_ site coordination microenvironment of a truncated acetyl-coenzyme A synthase, containing the C-terminal 136 residues of ACS (named ACS-α_15_) (Figure 1). Considering the difference between the coordination geometry of the Ni_d_ site and the oxidized Ni-SOD, it is believed that the axial ligand is indispensable for the stabilization of Ni^3+^ ions. In our previous work, one mutant, F598H, was designed to introduce an axial histidine ligand to the first coordination sphere of the Ni_d_ site, and the mutant protein successfully exhibited Ni-SOD-like activity [17]. Based on the same strategy, two more mutants, S594H and S594H-GP, were also designed and studied herein (Figure 1). These mutant proteins successfully exhibited Ni-SOD-like activity, although the catalytic activity was about ~10-fold less than that of the native Ni-SOD [18,19]. Based on structural analysis, the low SOD activity of the mutants is mainly due to lack of a favorable hydrogen bond network in the second sphere of the Ni_d_ site, although there are many factors that affect SOD activity. The X-ray structure of native Ni-SOD indicates that Tyr9 is involved in hydrogen-bonding with two ordered water molecules in the active site, a common feature in other SODs, which is believed to play a key role in regulating anion access [14,20]. Therefore, to better replicate the activity of the native Ni-SOD, the second step of our strategies was to mimic the H-bond network in the second sphere coordination microenvironment of Ni-SOD active site. Two more mutants were then designed and prepared based on the scaffold of F598H (EFG-F598H and YGP-F598H) (Figure 2), and especially the EFG-F598H mutant exhibited a ~3-fold increased Ni-SOD-like activity of F598H.

These designed Ni-SOD-like metalloenzymes were characterized by UV/Vis, EPR and Cyclic voltammetry. SOD-like activities were analyzed and the structural features responsible for the extraordinary SOD catalytic efficiency were elucidated by pH titrations and Electro Spray Ionization-Mass Spectroscopy (ESI-MS). The crystal structure of F598H, which contains an inter-molecular disulfide bond, was resolved to explain the impaired SOD activity caused by cysteine oxidation. Finally, the proposed molecular mechanism for the Ni-SOD-like metalloenzymes was presented.

## 2. Results

### 2.1. Design of Nid-Site by Molecular Modeling

To design and regulate the coordination microenvironment of the Ni_d_ site, an axial Histidine ligand was rationally introduced to the Ni_d_ site of ACS-α_15_ based on computer simulation, and the mutant protein, F598H, was successfully shown to have a Ni-SOD-like activity in our previous work [17]. To systematically investigate the influence of the axial His ligand on the Ni-SOD function, several axial His ligands in different positions were introduced to the Ni_d_ site in this work. One is S594H, which replaces the S594 with a His on the basis of ACS-α_15_, and the other is S594H-GP, which inserts two amino acids (Gly and Pro) between His594 and Cys595 on the basis of S594H. Molecular dynamics simulation showed that replacement of Ser594 by His could place the N_δ_ of His at ~2.71 Å from the nickel ion (Figure 1). On the other hand, by inserting a dipeptide Gly-Pro, S594H-GP could place the N_δ_ of His at 2.76 Å from the Ni center in the opposite direction compared to S594H and F598H (Figure 1). These distances between the N_δ_ of axial His ligand and the Ni ion are similar to F598H (2.73 Å) and the native Ni-SOD (2.67 Å) [16]. In the native Ni-SOD active center, there is a key residue Tyr 9 near the vacant coordination site and on the opposite side of the His1 axial ligand, which is thought to play a key role in the catalysis by providing an H-bond to help in positioning the O_2_^−^ substrate [20]. Therefore, to better replicate the secondary coordination environment of the nickel ion, two additional mutants, YGP-F598H and EFG-F598H, were designed based on the scaffold of F598H (Figure 2). YGP-F598H refers to a mutant on the basis of F598H, which replaces the S594 with Tyr and then inserts a dipeptide, Gly-Pro, between Try594 and Cys595. EFG-F598H means a variant of F598H, in which a tripeptide, Glu-Phe-Gly, was inserted into the position between Ser594 and Cys595. Molecular modeling studies showed that the introduced Tyr in YGP-F598H lies on the opposite site of axial ligand His598, and is located near the sixth coordination position of the Ni site with a Tyr-O-Ni distance of 5.32 Å, which is similar to the distance of Tyr9-O-Ni in native Ni-SOD (5.47 Å) [14,16]. In the case of EFG-F598H, the carboxyl group of the introduced Glu locates almost in the same plane with the square-planar [NiN_2_S_2_] center with a Glu-O-Ni distance of 6.87 Å. (Figure 2).

### 2.2. Mutagenesis, Expression, and Purification

The F598H variant and all the other derivatives contain the C-terminal 136 residues of ACS, thus apparently retain both the Nip and Nid components, but lack the N-terminal [Fe4S4] cluster. To simplify the purification process and avoid unnecessary amino residues remaining in the N-terminus, a smt3 tag (small ubiquitin-like modifier tag 3) gene was inserted into the position between the His tag and F598H gene. Then, according to the design, the genes of ACS-α_15_, S594H, S594H-GP, EFG-F598H and YGP-F598H with a smt3 tag were constructed by site-directed mutagenesis. All the mutant proteins were efficiently overexpressed with His-smt3 tags in *E. coli*. After the SUMO protease cleavage, quantities of the mutant proteins with more than 95% purity were obtained successfully (Figure S1). All the mutant proteins exhibited a green color similar to F598H after reconstitution with NiCl_2_ [17]. Metal analysis indicated that Ni was the only first row transition metal of significant concentration (Table 1). Gel filtration analysis indicated that all four mutants were present mainly as homodimers following reconstitution with NiCl_2_, which is similar to ACS-α_15_.

### 2.3. Nickel Binding Study by UV-Vis and ITC

**Figure 3:**
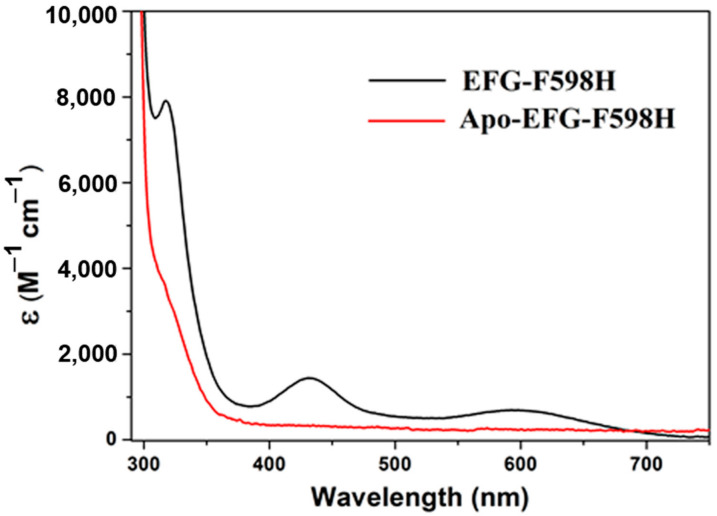
UV-Vis spectra of apo-EFG-F598H (red) and Ni reconstituted EFG-F598H (black).

Electronic absorption spectra of apo- and Ni-reconstituted EFG-F598H are presented in Figure 3 as a representative. The absorption peaks of S594H, S594H-GP and YGP-F598H mutants were extremely similar to EFG-F598H, and almost identical to F598H (Figure S2). Extinction coefficients are summarized in Table 1. The lower energy transitions (430 nm and 590 nm) have extinction coefficients consistent with d-d transitions, and the higher energy transition (317 nm) is most consistent with a transition of charge transfer [21].

The electronic absorption around 590 nm can be assigned to the nonbonding Ni(3dz^2^) to Ni(3dx^2^ − y^2^)/S/N(σ)* transition, and the one around 430 nm is due to a Ni(3dxy)/S(π)* to Ni(3dx^2^ − y^2^)/S/N(σ)* transition, which are characteristic of square-planar [NiN_2_S_2_] complexes. Meanwhile, the transition around 317 nm might be derived from LMCT or an electron transition into the orbital that has higher energy than LUMO. Although it had not been reported before, the absorption around 317 nm also exists in F598H. These UV-Vis spectral properties indicated that the coordination environment of the Ni ion in all of these mutants was not significantly perturbed [14]. The coordination environment of the Ni ion in EFG-F598H was further supported for Ni binding in the right environment by Isothermal Titration Calorimetry (ITC). The calorimetric titration result for titrations of 1.0 mM NiCl_2_ into a solution of 50 μM protein of apo-EFG-F598H are shown in the supporting information (Figure S3). The data correction and curve fitting were performed using Origin 7.0. The curve fit with a one-site sequential binding model well and the Ni^2+^ affinity constants (*K_d_*) of EFG-F598H is 6.4 ± 0.5 μM (Figure S3).

### 2.4. Electron Paramagnetic Resonance

**Figure 4:**
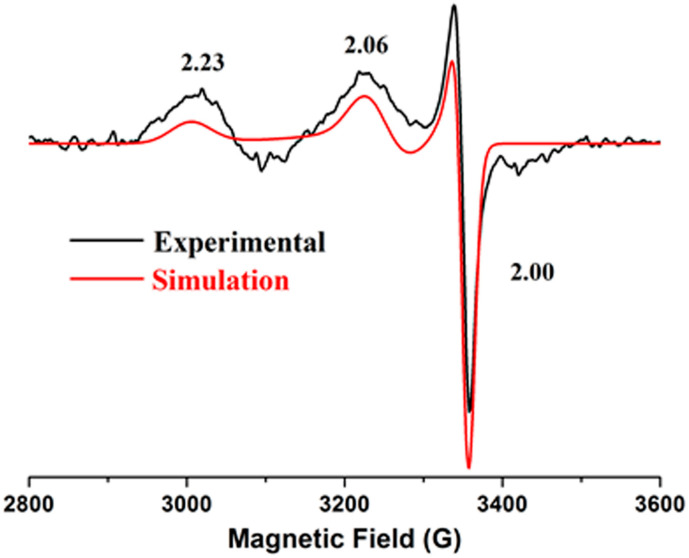
X-band EPR spectrum of EFG-F598H as a representative at 2 K in the presence of ~1 equiv of KO_2_ (microwave frequency, 9.49 GHz; microwave power, 20 mW; modulation amplitude, 5 G). The red line was obtained by simulation.

EPR spectroscopy was used to examine the redox state of the nickel center and probe the electronic structure of the Ni site. Since all the isolated mutants were in a reduced Ni^2+^ state, ~1 equivalent of KO_2_ was added to trap the oxidized Ni^3+^ intermediate. The 2 K EPR spectra of S594H, S594H-GP, EFG-F598H and YGP-F598H were exactly the same and displayed a rhombic S = 1/2 Ni^3+^ signal with g_x_ = 2.23, g_y_ = 2.06, and g_z_ = 2.00 (Table 1), indicating a five-coordinate, low-spin Ni^3+^ center (Figure 4 and Figure S4). These features are similar to those observed in F598H [10], Ni-SOD_ox_ [16,21,22], and a few synthetic square-pyramidal small molecule complexes [16]. The EPR spectra also revealed a small anisotropic signal at g_x_ = 2.08, g_y_ = 2.00, and g_z_ = 2.00, which originated from excess O_2_^−^ radical [23]. The expected super hyperfine coupling along g_z_ was not detected, which might be due to weak hyperfine interaction between the Ni^3+^ and the N_δ_ of axial His with a relatively long bond distance or was covered by the O_2_^−^-radical signal [24,25] (Figure 4).

The EPR data indicated that all these mutants could stabilize the Ni^3+^ intermediates upon oxidation. The signal of S594H was stronger than F598H and similar to Ni-SOD, which might be caused by the smaller distance between His-N_δ_ and Ni center. Meanwhile, the EPR spectra of EFG-F598H and YGP-F598H were extremely similar to F598H, which indicated that the electronic structures of the Ni^3+^ centers were not significantly perturbed by mutations in the second coordination sphere.

### 2.5. Cyclic Voltammetry

**Figure 5:**
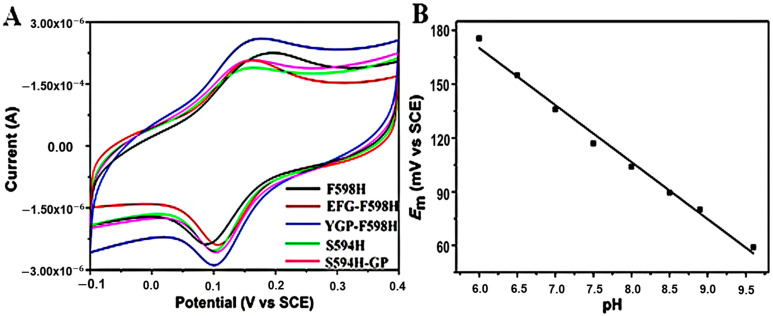
(**A**): Cyclic Voltammograms at MPA-modified Au electrode in 100 mM PBS (pH = 7.0) containing F598H (black), EFG-F598H (red), YGP-F598H (blue), S594H (green) and S594H-GP (pink). Potential scan rate: 100 mV/s; (**B**): Plots of the formal potentials of EFG-F598H as a representative at the MPA-modified Au electrode vs. solution pH.

To determine the redox potential of Ni^2+^ to Ni^3+^ transition in the mutants, direct electrochemical measurements were performed using cyclic voltammetry (CV), which is sensitive to the ligand-field environment. A gold electrode modified with a self-assembled monolayer of 3-mercaptopropionic acid (MPA) was used as a working electrode to promote the direct electron transfer [26]. CV curves of S594H, S594H-GP, EFG-F598H and YGP-F598H obtained in a 100 mM phosphate buffer solution (pH 7.0) displayed quasireversible behaviors for the Ni^2+/3+^ redox couple at ~375 mV vs. NHE (Normal Hydrogen Electrode) (Figure 5A and Table 2). The small peak separation of ~70 mV is indicative of one-electron redox progress occurred at the Ni center. The *E*_m_ values are about 200 mV lower than that of F598H reported before (570 mV) [17], and are closer to that of Ni-SOD (290 mV) [20] and the midpoint (365 mV) for redox couples of O_2_/O_2_^−^ (−160 mV) and O_2_^−^/H_2_O_2_ (890 mV) [27]. It should be noted that the large different *E*_m_ values from F598H may be caused by the different methods used to modify the working electrode. *N*,*N*-dimethyldioctadecylammonium bromide (DDAB) used in previous work may cause F598H partial denaturation. Therefore, the potential of F598H used an MPA-modified gold electrode was redetermined to be 372 mV vs. NHE, which was consistent with the other mutants within the error range.

The redox potentials of S594H and S594H-GP were extremely similar to F598H, indicating that all the mutants that introduce an axial ligand have suitable *E*_m_ values to catalyze the superoxide disproportionation. Moreover, the *E*_m_ values for EFG-F598H and YGP-F598H are also similar to F598H, which indicated that the mutations cannot significantly influence the electron transfer between Ni^2+^ and Ni^3+^. These results agree with the observation for Mn-SOD and Ni-SOD, in which mutations of Tyr34 and Tyr9 do not affect the redox potential of the Mn and Ni center [20,28].

In addition, the midpoint potential of EFG-F598H was found to be dependent on the pH value of the solution (Figure 5B). It decreased linearly with the increasing pH, with a slope of ~30 mV/pH from pH 6.0 to pH 10.0, indicating two protons and one electron were involved in the electrode reaction of EFG-F598H, which can be characterized by the half-reaction ENi^3+^ + e^−^ + 2H^+^ = H^+^_2_ENi^2+^ [29]. This observation provides evidence for coupled electron/proton-transfer reactions at the active site [30].

### 2.6. SOD Activity

**Figure 6:**
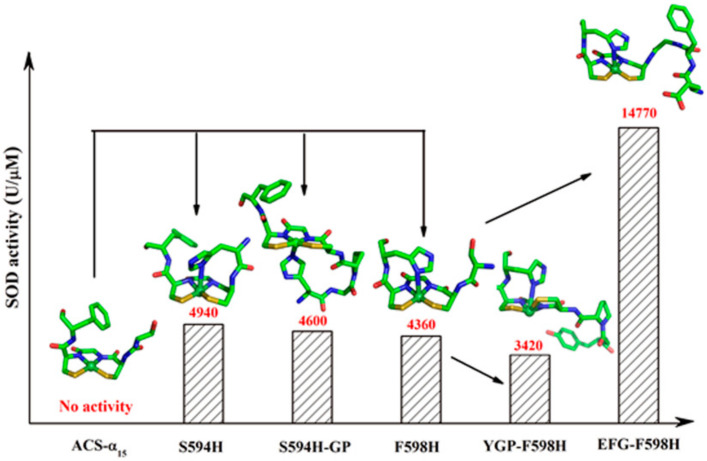
SOD-like activity with structures of the truncated ACS protein ACS-α15 (PDB: 3S2X) and modeling structures of S594H, S594H-GP, F598H, YGP-F598H and EFG-F598H.

One focus of this study is to investigate whether the activity of the mutants was regulated by the designed microenvironment of the Ni site. Table 2 summarizes the catalytic rate constants (Figure S5) for all these mutants [20]. S594H and S594H-GP display a little higher activity than F598H. EFG-F598H exhibits a considerably enhanced activity, which is ~3-fold higher than that of F598H, while ~3-fold lower than that of native Ni-SOD from *S. coelicolor* (45,292 U/μM) [18,31]. Meanwhile, the catalytic activity of YGP-F598H is only ~80% of F598H (Table 2 and Figure 6). These results demonstrated that the residues designed to form a second coordination sphere are intimately involved in the catalytic reaction.

**Table 2:** *E*_m_ and SOD activity (pH 7.5) of ACS-α15 Mutants.

Sample	No. of Ni Atoms per Protein	*E*_m_ (mV vs. NHE)	SOD Activity (U/μmol)
F598H	0.7	372	4360
S594H	0.8	374	4940
S594H-GP	0.8	375	4600
EFG-F598H	0.6	377	14,770
YGP-F598H	0.5	374	3420

### 2.7. Effect of pH on SOD Activity

Since pH can influence the visible absorption of SOD [32,33], its effects on the designed Ni-SOD-like enzymes were observed. The pH-dependent absorption changes at 430 nm are shown in Figure S6. The Henderson-Hasselbalch equation for a single p*K* was used to fit the curves. All the p*K*_a_ values were determined to be ~7.3, which was believed to be identical within experimental error. As 430 nm is defined as characteristic absorption of square-planar [NiN_2_S_2_] complexes, the changes could be caused by the protonation of His598-N, Cys595-S/Cys597-S or H_2_O bonded around the Ni center. As ACSα_15_, which lacked the axial histidine ligand, displayed a similar p*K*_a_ to other three mutants, the p*K*_a_ at 7.3 can be attributed to the protonation of Cys595/Cys597 or H_2_O.

**Figure 7:**
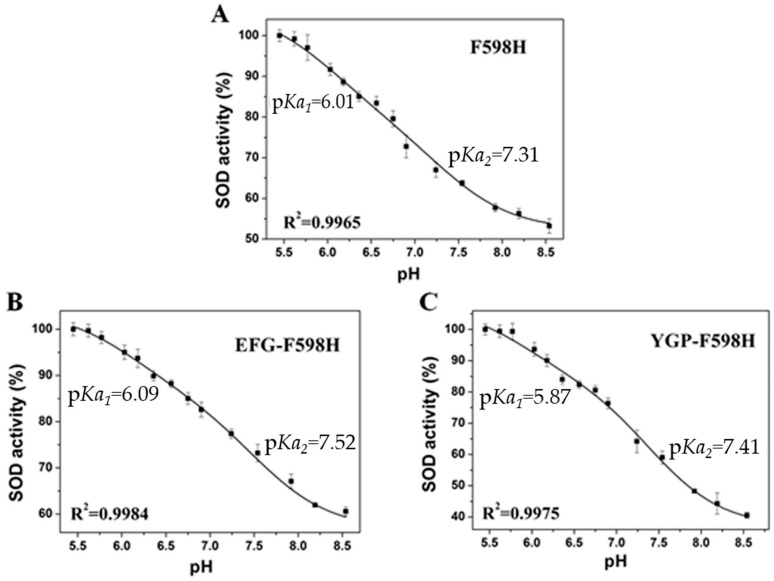
The effect of pH changes on the rate constants for the enzyme reaction of F598H (**A**), EFG-F598H (**B**), and YGP-F598H (**C**).

As reported previously, the activity of native Ni-SOD is pH dependent, which is similar to other SODs, particularly Mn-SOD [27,32]. In this study, the rate constants of F598H, EFG-F598H and YGP-F598H were determined at different pH values. The catalytic rate constants changed as a function of pH revealed two pH transitions, with p*K*_a_s equal to ~6.0 and ~7.4 (Figure 7). According to the result of direct electrochemistry, the reduced enzymes are believed to supply two protons for the reaction to form H_2_O_2_. Therefore, the p*K*_a_s observed in the SOD activity measurements can be attributed to the protonation/deprotonation of the ligands that supply protons for the reaction. As we know, the p*K*_a_ value of free histidine is 6.04, which is consistent with the observed lower p*K*_a_ value. So, one of the protons required for the catalytic cycle probably comes from His598, which can coordinate to the oxidized Ni center and does not interfere with the absorption spectra of the reduced Ni center. Besides, the similarity between the higher p*K*_a_ in SOD activity measurements and the optical p*K*_a_ suggests that these two values correspond to the same event. Hence, the second proton may come from Cys595/Cys597 or H_2_O bonded around the Ni center. This result is different from the previous reports of Ni-SOD, which always has one p*K*_a_ [27]. However, the different Ni center structure and different mechanism of protons transfer may rationalize these observations.

Moreover, the secondary structure alteration of EFG-F598H induced by pH changing was examined by monitoring the ellipticity at 222 nm of circular dichroism spectra. As shown in Figure S7, no apparent change in ellipticity values was noticed across a pH range of 5 to 10, which indicated pronounced stability of EFG-F598H against pH changing. This result proved that the pH-dependent changes observed above were indeed caused by the protonation/deprotonation of the His, Cys or H_2_O coordinated to the active-site nickel, which provides clues for underlying dismutation mechanism of these NiSOD-like enzymes, especially for the protons’ source.

### 2.8. Electro Spray Ionization-Mass Spectroscopy (ESI-MS)

It was reported that a bisamidate donor within [Ni^2+^N_2_S_2_] complexes would lead to relatively rapid thiolate ligand oxidation under aerobic conditions and yield a terrible defective activity [24,34,35]. In order to find out the relationship between the inner sphere coordination environment and defective activity, ESI-MS, a powerful analytical technique allowing quantitative analysis of S-oxidated-proteins, was performed. All the mutants were expressed and purified under aerobic conditions, and the ESI-MS analysis showed that the measured molecular weight of EFG-F598H is 15,054.9 Da, which is consistent with the theoretical value (15,057.3 Da), indicating no evidence of oxidative modification. In stark contrast, the measured molecular weights of F598H and YGP-F598H are 14,694.3 Da and 14,925.8 Da, respectively, which are ~32 Da greater than the theoretical value (14,666.9 Da and 14,897.2 Da) (Figure S8). This phenomenon suggests that aerobic conditions can yield extensive oxidative modification to these two mutants. The extra 32 Da is due to the insertion of two oxygen atoms, corresponding to the oxidization of one thiolate ligand to a coordinated sulfinate, as in the metallopeptide-based mimics [35].

These observations suggest that EFG-F598H can protect the nickel center from oxidation, whereas F598H and YGP-F598H cannot, which may rationalize that EFG-F598H has a relatively higher catalytic activity.

### 2.9. Crystal Structure of F598H

In the course of the study, it was found that F598H purified under aerobic conditions without the reductant had a lower activity than those purified in the presence of the reductant. To explain this phenomenon, non-reduced SDS-PAGE and gel filtration chromatography were performed to analyze the component of F598H. The non-reduced SDS-PAGE system does not contain any reductant that could destroy disulfide bonds, so that it can distinguish the components that contain inter-molecular disulfide bonds. The results showed that a small part of F598H was present in the form of dimer and even tetramer caused by inter-molecular disulfide bonds. When the reductant, Tris(2-carboxyethyl) phosphine (TCEP), was added to the protein, the tetramer of F598H disappeared, and the dimer also reduced (Figure S9A). The results of gel filtration chromatography were identical. F598H purified under aerobic conditions without the reductant displayed a main peak for the dimer and accompanied by a shoulder, whose molecular weight was consistent with a tetramer F598H. However, F598H purified with reductant, which was verified to contain no inter-molecular disulfide bond by non-reduced SDS-PAGE, did not display the tetramer shoulder peak (Figure S9B). These results indicated that absence of the reductant indeed led to the formation of an inter-molecular disulfide bond, which might be the reason for the decreased activity.

**Figure 8:**
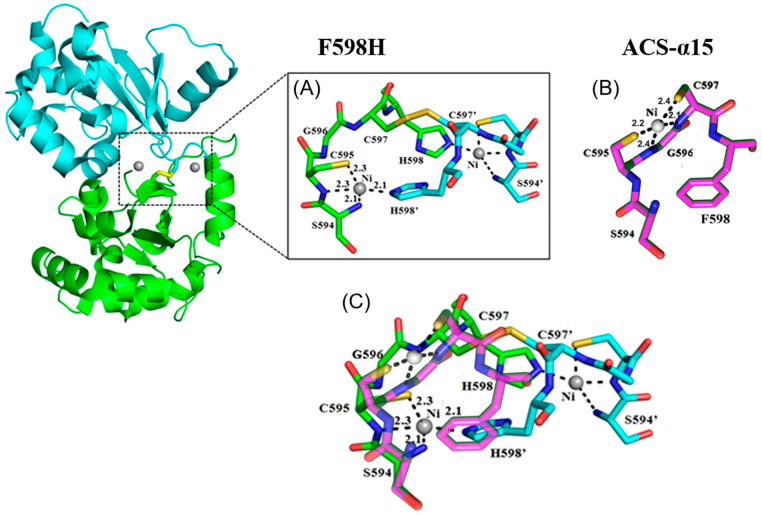
Overall structure of F598H (PDB: 5GOL) purified under aerobic condition and the nickel binding sites. (**A**) structure of the Ni metal center in F598H, Chain A colored by green, Chain B colored by cyan; (**B**) Ni metal center in ACS-α_15_ (PDB: 3S2X), colored by purple; (**C**) Superposition of F598H (green) and ACS-α_15_ (purple). Ni in grey, S in yellow, O in red and N in blue.

To confirm this result above, a 2.20 Å resolution structure of F598H that contained inter-molecular disulfide bond was determined under aerobic conditions. In the crystal structure (PDB: 5GOL), there were four asymmetric molecules. Each two molecules were connected by one disulfide bond to form a pair. Each pair of molecules contained two nickel metal centers. Two F598H subunits were found to form a dimer, while the nickel binding sites were located at the dimer interface. The structure diagram revealed that the two subunits interacted with each other through one disulfide bond (Figure 8). The structural alignment indicated that the overall structure of F598H was similar to ACS-α_15_ with a root-mean-square deviations (RMSD) of 0.36 Å, indicating that mutation of Phe598 to His598 did not affect the correct folding of F598H (Figure S10) [36].

In the “active center” of F598H, the only Ni atom was located at the Ni_p_* site as mentioned in ACS-α_15_ and was coordinated by one N-terminal NH_2_ (Ser594) with a distance of 2.1 Å, one backbone amide N atom of Cys595 (2.1 Å), one cysteine S atom of Cys595 (2.3 Å) and one imidazole N atom of His598 (2.3 Å) from another F598H subunit (Figure 8). However, the designed active metal center, which should be located on the Ni_d_ site, was absent. This must be due to the formation of an inter-molecular disulfide bond between two Cys597, which is a key residue coordinated to the Ni_d_ center. As the Ni_p_* center lacks the fifth axial ligand, it is also impossible to have any Ni-SOD activity. So, the crystal structure indicated a F598H without SOD activity, which could explain the impaired SOD activity.

## 3. Discussion

Based on the structural analysis, two obvious differences existed at the Ni-site between the truncated ACS-α_15_ and native Ni-SOD. The Ni_d_-site of ACS-α_15_ lacks axial histidine ligand in the nickel coordination geometry and is without the H-bond network in the coordination microenvironment. Histidine nitrogen deprotonation upon metal ion coordination can afford negative charges to stabilize metal ions in higher oxidation states (e.g., Ni^3+^ and Fe^3+^) [37,38,39]. Upon oxidation, the Ni^3+^ active site coordination geometry in Ni-SOD is converted from square-planar to square-pyramidal with His1 fifth coordination bond. It was shown that when the axial His ligand in the Ni-SOD was removed or substituted with Asp or Ala, the catalytic activity was impaired, which indicated the critical role of the axial His ligand [22,40]. However, the Ni_d_-site of ACS-α_15_ lacks an axial histidine ligand, therefore it remains in the Ni^2+^ state and the Ni^3+^ state cannot be captured even in the presence of strong oxidants. For functional conversion of ACS-α_15_ at the Ni_d_ site to a Ni-SOD-like enzyme, molecular designs have been implemented using molecular dynamic simulations. An axial histidine ligand was introduced by the mutations (F598H, S594H and S594H-GP individually), to the first coordination geometry of the Ni_d_ site. The resulting three mutant proteins were successfully converted into functional Ni-SOD-like enzymes, and the role of axial histidine ligand in stabilizing Ni^3+^ at the Ni_d_ site has been confirmed. UV-vis spectra of the mutants indicated that the square-planar coordination environment of the Ni_d_ site was not disturbed in reduced states. EPR spectra of the designed mutants testified that the axial ligand had the ability to form the fifth coordination bond and stabilize the Ni^3+^ ion. Moreover, S594H exhibited a more intensive EPR signal than F598H, which might be in line with its higher activity. Additionally, the suitable *E*_m_ values of the mutations provided further evidence for their Ni-SOD-like activity. However, the SOD activity of the designed mutants is lower than that of native Ni-SOD, which may be due in part to lacking the suitable H-bond network in the coordination microenvironment of the Ni_d_ site.

In native Ni-SOD, Tyr9 lies near the vacant sixth coordination position, and is in opposite position of the His1 axial ligand, with a Ni-O (Try9) distance of 5.47 Å. The phenol proton of Tyr9 is believed to involve the hydrogen bonds network with two ordered water molecules [14,16,27]. The Tyr 9 residue is thought to play a key role in the catalysis by providing a H-bond to regulate anion access [20]. Thus, to further improve the SOD activity and to study the catalytic mechanism of Ni-SOD based on these NiSOD-like enzymes, another two mutants, EFG-F598H and YGP-F598H, were designed, which better replicated the secondary sphere coordination microenvironment of the Ni_d_ site. As characterized by UV-Vis and EPR studies, F598H, EFG-F598H and YGP-F598H have very similar UV-Vis and EPR features, which indicated that introducing a hydroxyl around the metal center does not disturb the structure of the Ni_d_ site in either the reduced or oxidized state. Moreover, extremely similar potentials were obtained for these mutants by direct electrochemical measurement, which indicated that the mutations do not significantly influence the electron transfer in the redox process required for catalyzing the O_2_^−^ disproportionation reaction. Although these mutants have similar spectral properties and redox potentials, distinct SOD activities were exhibited. EFG-F598H has ~300% enhanced activity compared to F598H, whereas YGP-F598H only has 60% activity compared to F598H. These observations demonstrated that the residues introduced in the coordination microenvironment are intimately involved in the redox reaction.

To understand how these mutant enzymes participate in the disproportionation reaction and thus how they influence the activities, the catalytic mechanism was investigated. Until now, at least three controversies still exist in the catalytic mechanism of Ni-SOD: where the two protons needed for the reaction come from; if the substrate binds to the Ni center directly; and from which direction the substrate approaches the active center. Firstly, a series of pH titrations were performed to determine the two protons needed for the formation of H_2_O_2_. The results of CV titration indicated that two protons and one electron were involved in the electrode reaction, and the two protons should be supplied by residues around Ni_d_ center or some small molecule bonded around the Ni_d_ center, such as H_2_O. The results of UV-vis absorption titration inferred that one of the protons came from Cys595/Cys597 or H_2_O bonded around the Ni center. Besides, the results of SOD activity rate titration confirmed the conclusion of UV-vis absorption titration and also revealed that the other proton came from the axial histidine. This result is similar to a previous calculated report of Ni-SOD [41]. Secondly, to confirm if the substrate binds to the Ni center directly, anions such as azide ion and thiocyanide ion were added to the reaction system. SOD activity change was not found, which supported an outer-sphere redox reaction for both half-reactions. Lastly, the SOD activity of the mutants, in which negatively charged residue was introduced to the opposite site of the axial ligand, was analyzed to determine the attack direction of the substrates. As the results showed, the SOD activity of YGP-F598H (3420 U/μM) was lower than that of F598H (4360 U/μM), which might be due to the electrostatic repulsion between the introduced negatively charged residue and substrates. This phenomenon also existed in another two mutants, S594H-GP-F598Y (2100 U/μM) and S594H-GP-F598D (2740 U/μM) (Figure S11). So, it is believed that the substrates would attach the Ni site from the opposite direction of the axial ligand.

**Figure 9:**
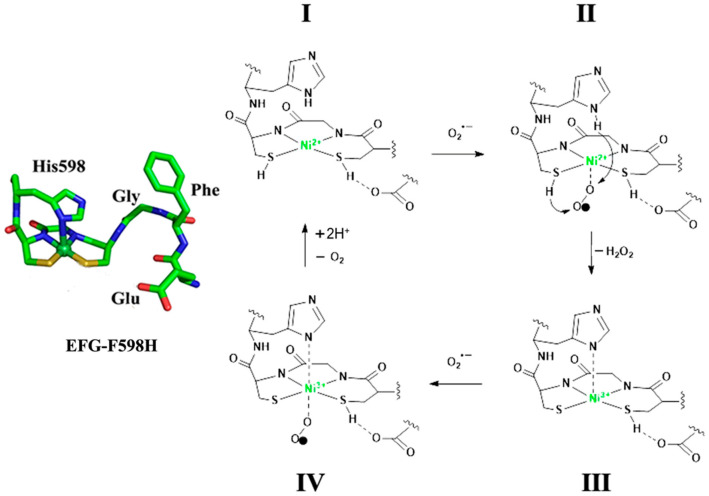
Proposed Outer-Sphere Catalytic Mechanism for EFG-F598H. State I: the begin of the reaction; State II: superoxide anion attacks the Ni site, the first half reaction; State III: His-on Ni^3+^ state; State IV: Superoxide also approaches the nickel center, the second half reaction.

Therefore, an outer-sphere catalytic mechanism for the Ni-SOD-like mutants based on Ni_d_-site of ACS-α_15_ is proposed (Figure 9). At the beginning of the reaction (State I), the mutant enzyme is in His-off Ni^2+^ state with protonated histidine and cysteine/H_2_O. The superoxide anion attacks the Ni site from the opposite site of the histidine axial ligand (State II). Then, electron transfer occurs concomitant with proton transfers, leading to formation of peroxide and His-on Ni^3+^ state with deprotonated histidine and cysteine/H_2_O. With the end of the first half reaction, the second half reaction starts immediately (State III). Superoxide also approaches the nickel center from the opposite position of the histidine axial ligand (State IV). With the transfer of the electron, an oxygen molecule and a His-off Ni^2+^ state are yielded. At the same time, the mutant proteins obtain two protons from the solvent, and protonated histidine and cysteine/H_2_O are formed again (State I). Hereto, a catalytic cycle is completed.

Although a mechanism for the Ni-SOD-like mutants based on the Ni_d_-site of ACS-α_15_ is proposed, the high SOD activity of EFG-F598H still cannot be understood well. Therefore, the active metal centers of the mutants and native Ni-SOD are analyzed again. Although ACS-Ni_d_ displays a similar square-planar coordination environment to reduced Ni-SOD, there is still a difference between these two sites. The nickel ion in Ni-SOD contains a mixed amine/amidate coordination motif, whereas the Ni_d_ site in ACS-α_15_ contains a bis-amidate coordination motif. It was reported that a bis-amidate donor within [Ni^2+^N_2_S_2_] complexes could lead to relatively rapid thiolate ligand oxidation under electrophilic attack by O_2_ and reactive oxygen species (ROSs) [35]. However, the combination of amine and amidate donors-set could also yield a stable nickel center under identical conditions, and such oxidation events could yield a terrible defective activity [35]. Despite these observations, the mutants of truncated ACS-α_15_ in this study still keep a relatively high activity, even though they all have bis-amidate ligands, which is different from that of the nickel-containing metallopeptide-based Ni-SOD mimics [24,34]. To address this issue, ESI-MS was used to analyze the mutant enzymes expressed and purified under aerobic conditions. As evidenced by ESI-MS, both F598H and YGP-F598H were extensively modified by insertion of two oxygen atoms. Similar to the observation for metallopeptide-based mimics, these results correspond to the oxidized of one thiolate ligand to a coordinated sulfinate [35]. Surprisingly, EFG-F598H has the MW expected for the amino acid substitutions involved, suggesting no thiolate ligand has been oxidized under aerobic conditions, which is consistent with a higher catalytic activity. As EFG-F598H and F598H are identical in the inner sphere coordination, the protective effect of the thiolate ligand might be due to the effect of the second coordination sphere, hydrogen bonding and/or steric interactions. Molecular dynamic simulation showed that the glutamate introduced in EFG-F598H was flexible and the distances between the Glu-O and Cys595/597-S were 4.62/6.46 Å, which was most likely involved in the hydrogen bonds network with the protonation of thiolate ligand. However, YGP-F598H cannot protect the thiolate ligand from oxidation, which might be attributed to inappropriate steric interactions. The phenol oxygen of Tyr in YGP-F598H is far away from both the sulfur atoms with distances of ~7 Å, and because of the presence of proline, the tyrosine is not as flexible as glutamate in EFG-F598H. Therefore, it is difficult for YGP-F598H to form an H-bond with cysteine.

Besides, another situation that can affect the activity of the Ni-SOD-like enzymes was observed. When the reductant is absent in the purification system, a small part of the key cysteine residues that should coordinate to the active Ni center will be oxidized to form inter-molecular disulfide bonds. The results of crystal structure indicated that these disulfide bonds lead to the deficiency of the active Ni_d_ center, and thus cause the loss of Ni-SOD activity. Even so, Ni-SOD-like enzymes still have enhanced catalytic activities compared with small molecules and metallopeptide Ni-SOD mimics, which should be attributed to the protective effect on the active Ni center and the stereo regulation of the substrate access by the protein side chain.

## 4. Materials and Methods

### 4.1. Materials

Pfu DNA polymerase, T4 DNA ligase, dNTP, and restriction enzymes (Nde I, and Xho I) were purchased from New England Biolabs. The KOD-Plus Mutagenesis Kit was purchased from TOYOBO. The *E. coli* strains Trans 10 and BL21(DE3) PlysS were obtained from TransGen. The plasmid purification kits, gel extraction kits, nickel nitrilotriacetic acid resin and Sephadex G-25 resin were purchased from QIAGEN (Chatsworth, CA, USA). The SuperdexTM 200 HiLoad 16/60 gel filtration column was from Pharmacia. The xanthine, xanthine oxidase and NBT were obtained from Sigma (St. Louis, MO, USA). The KO_2_ was purchased from Acros. All other reagents were of analytic grade.

### 4.2. Cloning, Expression, and Purification

F598H was cloned similarly as described [17], but the cloning detail method was modified by inserting a smt3 gene tag between the His tag and F598H. The F598H variant and all other derivatives contain the C-terminal 136 residues of ACS thus apparently retain both the Nip and Nid components, but lack the N-terminal [Fe_4_S_4_] cluster. Overlap extension PCR was used. In the first round of PCR, the primers P1 and P2 were used to amplify the smt3 and F598H genes, respectively. Then the purified PCR products were used as the templates in the second round PCR, using primers P1-F and P2-R. The resulting PCR product digested with *Nde* I and *Xho* I was inserted into a pET-28b (+) vector. Based on the His-smt3-F598H template, EFG-F598H and YGP-F598H were constructed by the KOD-Plus Mutagenesis Kit (TOYOBO, Osaka, Japan), using P3 and P4. Using P5, the His-smt3-F598H was mutated back to His-smt3-ACSα_15_. Then S594H and S594H-GP were constructed using P6 and P7. Primer Pairs were listed in support information (Table S1). In each case, the cloned region of the plasmid was sequenced (JieLi biology, Shanghai Co., Ltd., Shanghai, China) to verify the fidelity of the PCR reactions.

The expression and purification of the mutant proteins were carried out according to the reported methods [17]. The only difference was that the His-smt3 tag was removed by SUMO protease. The imidazole in the elution was removed by dialyzing for three times at 4 °C. The His-smt3 fusion tag was cleaved upon incubation with SUMO protease for 8 h at 16 °C, and the smt3 tags and liberated proteins were separated re-subjected to Ni-NTA column. All proteins were found to be at least 95% pure based on the denaturing polyacrylamide gels stained with Coomassie Brilliant Blue (Figure S1).

### 4.3. Metal Reconstruction and Metal Analysis

The isolated mutant proteins had a little amount of Ni^2+^, and the nickel reconstitution of the mutants was carried out by incubation with NiCl_2_. The Ni-activated proteins were subjected to a G-25 column to remove excessive nickel ions. The Ni-activated proteins were dialyzed three times by dialyzing, respectively, in the presence of 5 mM 10-phenanthroline (phen) in buffer for 2 h to remove the labile Nip in the Ni-reconstructed mutant proteins. The excessive phen and [Ni(phen)_2_]Cl_2_ in the protein solution were removed by G-25 column.

Protein samples for metal analysis were digested with metal-free nitric acid and heated overnight at 65 °C. The denatured samples were diluted with metal-free water to be analyzed on a Pekin-Elmer Optima 3000 DV inductively coupled plasma optical emission spectrometer (ICP-OES).

### 4.4. Protein Characterization

UV-vis spectra were recorded on a HP 8453 UV/Vis spectrophotometer using a quartz cuvette with 1 cm path length at 25 °C as reported previously [10]. Low-temperature EPR spectra were recorded with a Bruker EMX X-band spectrometer equipped with an Oxford-910 cryostat and ITC-503 temperature controller (Oxford Instruments Ltd., Oxfordshire, UK). All data were analyzed with the Bruker WinEPR software. EPR settings: temperature 2 K, microwave frequency 9.49 GHz, microwave power, 20 mW, modulation amplitude 5 G. In general, Ni^3+^ species of the mutant proteins was generated in a 3:1 mixture of buffer solution: glycerol (buffer solution: 50 mM potassium phosphate, pH 7.4) in a quartz EPR tube [17]. ~1 equivalent of KO_2_ (dissolved using 18-crown-6-ether in DMSO) was added to a ~400 μM protein solution, and the mixture was quickly mixed in a vortex mixer and then immediately frozen in liquid nitrogen. The solvent system was checked carefully as controls, and no obvious signals were obtained. EPR simulation was performed with the EasySpin software.

### 4.5. Cyclic Voltammetry Assay

Cyclic voltammetry was performed on a CHI 660A electrochemical workstation (Chenhua, Shanghai, China). A standard three-electrode cell was utilized with a saturated calomel reference electrode (SCE, Kowloon Tong, Hong Kong, China), a Pt wire auxiliary electrode, and a protein/MPA-modified Au disk working electrode (0.2 cm^2^). MPA-modified Au electrodes were prepared using a previously demonstrated procedure [26]. The freshly prepared Au electrodes were soaked in a 20 mM MPA solution for 1 h. The electrodes were then rinsed with ethanol to remove the no chemisorbed MPA prior to use in electrochemical experiments. NiSOD-like enzyme/MPA-modified Au electrodes were prepared by soaking the MPA-modified Au electrodes in 0.1 mM mutant proteins for 6 h. The as-prepared electrodes were then rinsed with water and stored at 4 °C when not in use. The supporting electrolyte solution was 0.1 M phosphate buffer solution (PBS) pH 7.0. Cyclic voltammetry was recorded at a scan velocity of 100 mV/s. All potentials were cited versus the normal hydrogen electrode (NHE) using a correction of +242 mV for the potential of the reference electrode.

### 4.6. SOD Activity Assay

SOD activity assays were performed according to the McCord–Fridovich method using NBT. The xanthine/xanthine oxidase system was used as enzymic source for the generation of O_2_^−^ in this system. The experiment was performed in a 2 mL system as reported [17]. The absorption was recorded over a period of 60 s at 560 nm, and carred out every 5 s in a kinetic model on HP 8452 UV/Vis spectrophotometer. The SOD-activity (U) is defined as the half-limited reduction of NBT and measured by a reduction in the slope and set relative to the concentration of the substance: U = 2 (M_control_ − M_substrate_)/M_control_, where U is the activity unit of the metalloprotein relative to the amount present in the test (given in U/μmol). As the mutants contain different ratios of nickel, the finial SOD-activity was calculated according to the amount of Ni-containing protein.

### 4.7. Molecular Dynamic Simulation

The initial structures of these designed mutants were generated based on the X-ray structure of ACS-α_15_ (PDB: 3S2X) by adding the additional amino acids to the N-terminus using program VMD 1.9 (Visual Molecular Dynamics). The conformation of [Ni^2+^N_2_S_2_] and N_His_-Ni coordination were defined according to the X-ray structure of native Ni-SOD, with an average bond length of S-N, 2.24 Å, N-Ni (in plane), 2.00 Å, and N-Ni (in axial), 2.67 Å, respectively (PDB: 1T6U). Other parameters were obtained from the classical force field CHARMM27. The psfgen program of NAMD2.8 (Nanoscale Molecular Dynamics) was used to add hydrogen atoms and assign charges to the protein, which was set up according to pH 7.0. The protein was then solvated in a cubic box of TIP3 water with periodic boundary conditions, which extended 10 Å away from any given protein atom. The protein–water system was first minimized for 5000 steps (1 fs per step) using program NAMD 2.8 with conjugate gradient method at 0 K, subsequently equilibrated for 10,000 molecular dynamics steps via an NVT ensemble (where the number of particles N, the volume V, and the temperature T of the system were kept constant) at 300 K, a procedure similar to model F598H mutant in our previous study. The system was further minimized for 30,000 steps before analyzing it with VMD 1.9.

### 4.8. pH Titration Assay

Optical and activity pH titrations were performed over a pH range of 5 to 9 using 20 mM PBS buffer. The absorption intensity was recorded at the wavelength of 430 nm for optical pH titration. Before spectrophotometric measurements, each mutant was allowed to incubate in buffer of desired pH for 1 h at room temperature to reach equilibrium. The absorbance’s dependences on pH were fitted using the Henderson–Hasselbalch equation.

### 4.9. Isothermal Titration Calorimetry (ITC) Assay

To measure the nickel ion affinity of the F598H variant protein EFG-F598H, Isothermal Titration Calorimetry (ITC) was applied (MicroCal ITC 200, GE healthcare Life Sciences, Pittsburgh, PA, USA) at 25 °C. The protein sample (apo-EFG-F598H, 50 μM) was titrated against 20 mM HEPES buffer (pH 7.4) containing 1 mM NiCl_2_. Titrations were performed as 25 injections (1.5 μL each) of a metal stock solution into a protein solution in the sample cell (200 μL) of the calorimeter. Each metal injection required a 120 s relaxation interval between successive injections. The data correction and curve fitting were performed using Origin 7.0.

### 4.10. Electro Spray Ionization-Mass Spectroscopy (ESI-MS) Assay

ESI-MS was performed on an LTQ Orbitrap XL mass spectrometer (Thermo Electron Corp., Bremen, Germany). The purified proteins were subjected to a G-25 column to change the buffer solutions to water and 0.1% formic acid was added to the protein solutions just before the test. The protein concentrations were 5 mg/mL. The spray voltage was set at 1.6 kV and the heated capillary at 200 °C. The mass spectrometer was operated in positive mode at the mass range 350~1800 Da with resolution of 60,000. The AGC expectation during full-MS was 1,000,000. The error for the calculated mass is about ±5 Da.

### 4.11. Crystallography of F598H

F598H (~20 mg/mL) was crystallized at 16 °C with Tris·HCl (100 mM, pH 9.0)/(NH_4_)_2_SO_4_ (1.8 M) with a protein/reservoir solution ratio of 1:1 in the crystallization drops. Crystals were flash frozen in liquid nitrogen in the reservoir solution supplemented with glycerol (20%, *v*/*v*). The crystals diffracted to 2.20 Å at beam line BL17U at Shanghai Synchrotron Facility (SSRF). The nickel absorption edge was determined to be at 8343 KeV by fluorescence scanning. Diffraction data at the nickel peak wavelength (1.486 Å) were collected with one-degree oscillation widths through a range of 360°. The diffraction data were integrated and scaled by using the HKL2000 package [42]. The nickel atom was determined with the program SHELXD. Then the PHENIX software package was used to solve the structure. Briefly, the Phenix AutoSol was used to refine the locations of substructures, calculate the initial phases, make Density Modifications and build the initial model. After model built using Phenix AutoBuild, 125 of the total 135 residues were successfully built in the initial model. The final model was manually completed by the program Coot and further refined by Phenix Refine. The quality of the models was checked using PROCHECK [43]. A summary of data collection and refinement statistics are shown in Table S2. Atomic coordinates and structure factors have been deposited in the Protein Data Bank (PDB: 5GOL). The structural figure was prepared using PyMOL.

## 5. Conclusions

In summary, to functionally convert a truncated acetyl-coenzyme A synthase at Ni_d_-site to a Ni-SOD-like enzyme, we systematically modified the coordination microenvironments of the redox-inactive Ni_d_ site. The first strategy is to introduce an axial histidine ligand by mutation to the first coordination sphere of the Ni_d_ site. The resulting three mutant proteins obtained Ni-SOD-like activity successfully, although the catalytic activity was much lower than that of native Ni-SOD enzyme. The second strategy is to mimic the H-bond network in the coordination microenvironment of the Ni-SOD active site. A successful mutant EFG-F598H exhibited a ~3-fold increased Ni-SOD-like activity of F598H. The pH titrations revealed the source of two protons required for forming H_2_O_2_ in the SOD catalytic reaction. The crystal structure of F598H with an inter-molecular disulfide bond explained the impaired SOD activity caused by cysteine oxidation. Based on all the results, we proposed an outer-sphere catalytic mechanism for the Ni-SOD-like mutants, which provided a basis for the mechanism study of native Ni-SOD. Further studies are ongoing for enhancing the catalytic activity by improving substrate cavity with suitable hydrogen bond networks at the Ni-site coordination microenvironment.

## Figures and Tables

**Table 1:** UV-Vis and EPR data of ACS-α15 Mutants.

Sample	No. of Ni Atoms per Protein	Extinction Coefficient (M^−1^ cm^−1^)	EPR
		317, 430, 590 nm	
F598H	0.7	7760, 1495, 615	g = 2.23, 2.06, 2.00
S594H	0.8	7065, 1565, 640	g = 2.23, 2.06, 2.00
S594H-GP	0.8	7685, 1490, 615	g = 2.23, 2.06, 2.00
EFG-F598H	0.6	7905, 1440, 695	g = 2.23, 2.06, 2.00
YGP-F598H	0.5	7450, 1445, 590	g = 2.23, 2.06, 2.00

## Data Availability

The datasets for this manuscript can be obtained from the corresponding author upon reasonable request.

## Supplementary Materials

The following supporting information can be downloaded at: https://www.mdpi.com/article/10.3390/ijms23052652/s1.
