# Supplementary material for: Functional Conversion of Acetyl-Coenzyme a Synthase to a Nickel Superoxide Dismutase via Rational Design of Coordination Microenvironment for the Nid-Site
Source: Int J Mol Sci. 2022 Feb 28;23(5):2652. doi: 10.3390/ijms23052652 (PMC8910529; doi:10.3390/ijms23052652)
Supplement: Supplementary file 1 [file ijms-23-02652-s001.zip › ijms-1582997-supplementary.pdf]

International Journal of *Molecular Sciences*

MDPI

Supporting Information for

Functional Conversion of Acetyl-Coenzyme a Synthase to a Nickel Superoxide Dismutase via Rational Design of Coordination Microenvironment for the Ni_d-Site

Yaozhu Wei [1], Yajun Zhou [1], Hong Yuan [1], Yi Liu [1], Ying-Wu Lin [2], Jihu Su [3] and Xiangshi Tan [1,*]

[1] Department of Chemistry, Fudan University, Shanghai 200433, China; 12110220023@fudan.edu.cn (Y.W.); 15110220061@fudan.edu.cn (Y.Z.); yuanhong119@163.com (H.Y.); yiliu0825@hubu.edu.cn (Y.L.)

[2] School of Chemistry and Chemical Engineering, University of South China, Hengyang 421001, China; ywlin@usc.edu.cn

[3] Department of Modern Physics, University of Science and Technology of China, Hefei 230026, China; sujihu@ustc.edu.cn

* Correspondence: xstan@fudan.edu.cn

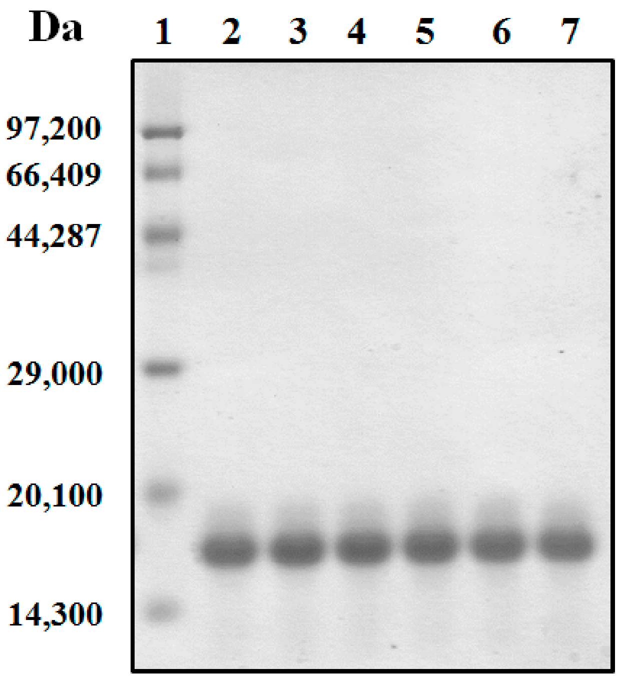

Figure S1. Sodium dodecyl sulfate polyacrylamide gel electrophoresis: Lane 1 marker, lane 2 ACS-α_{15}, lane 3 F598H, lane 4 S594H, lane 5 S594H-GP, lane 6 EFG-F598H, lane 7 YGP-F598H.

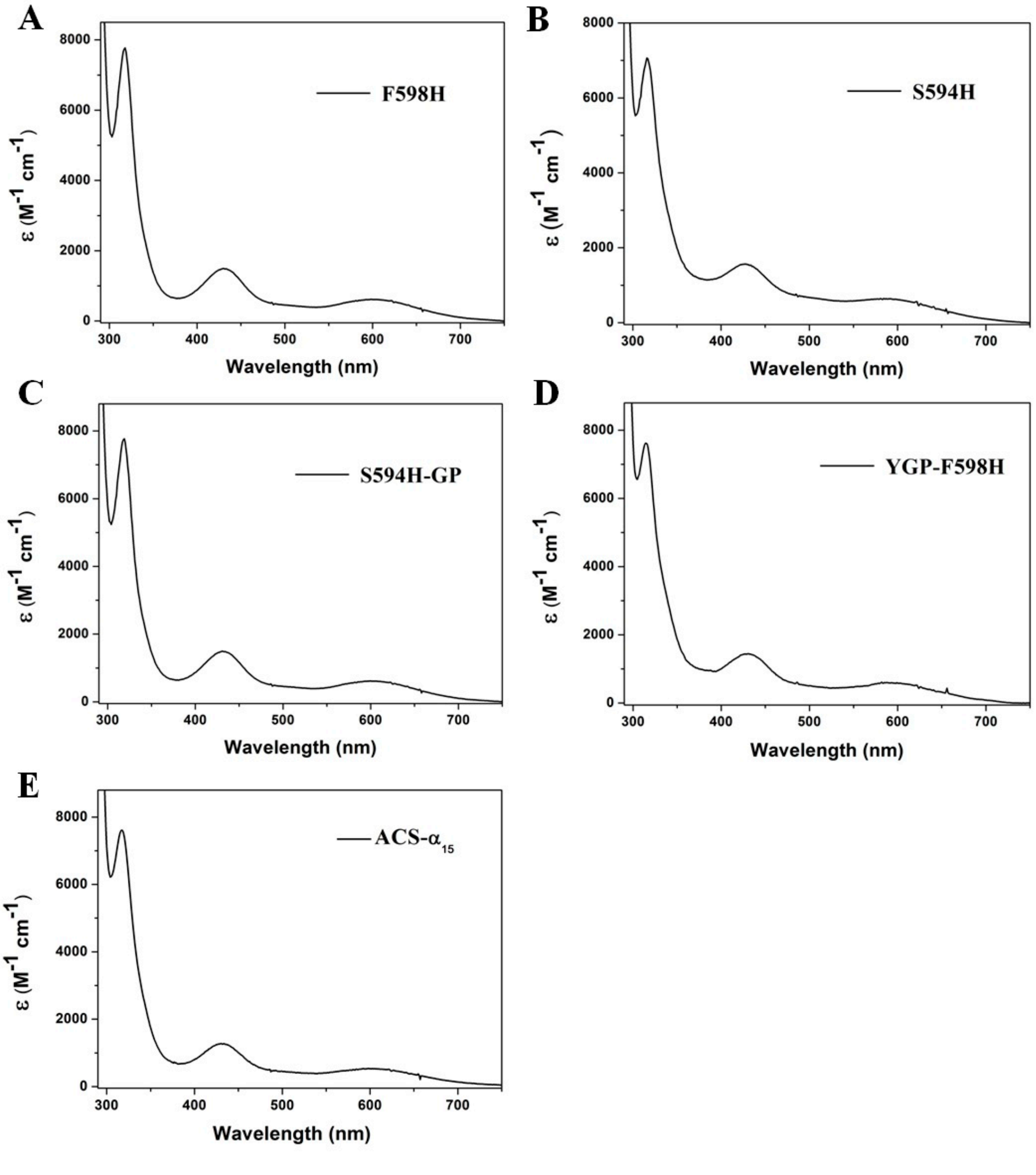

Figure S2. UV/Vis spectra of (A) F598H, (B) S594H, (C) S594H-GP, (D) YGP-F598H and (E) ACS-α_{15}.

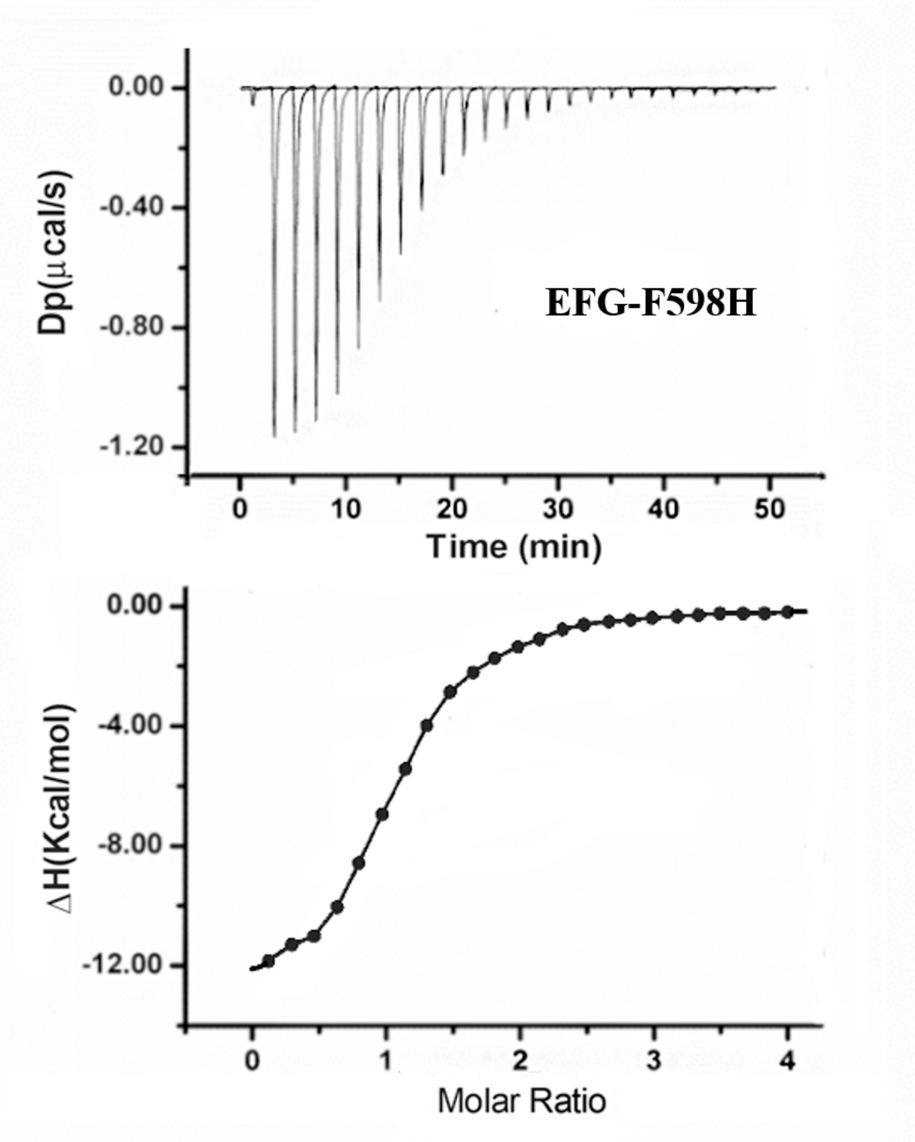

Figure S3. Calorimetric titration result for titrations of 1.0 mM $NiCl_2$ into a solution of 50 μM protein of apo-EFG-F598H. Curve fitted with a one-site sequential binding model. Protein sample (50 μM) was titrated against 20 mM HEPES buffer (pH 7.4) containing 1 mM $NiCl_2$. Titrations were performed as 25 injections (1.5 μl each) of a metal stock solution into a protein solution in the sample cell (200 μl) of the calorimeter. Each metal injection required a 120 s relaxation interval between successive injections. The data correction and curve fitting were performed using Origin 7.0. The ITC result for EFG-F598H was shown in Figure S7. Curve fitted with a one-site sequential binding model well and Kd = 6.4±0.5 μM.

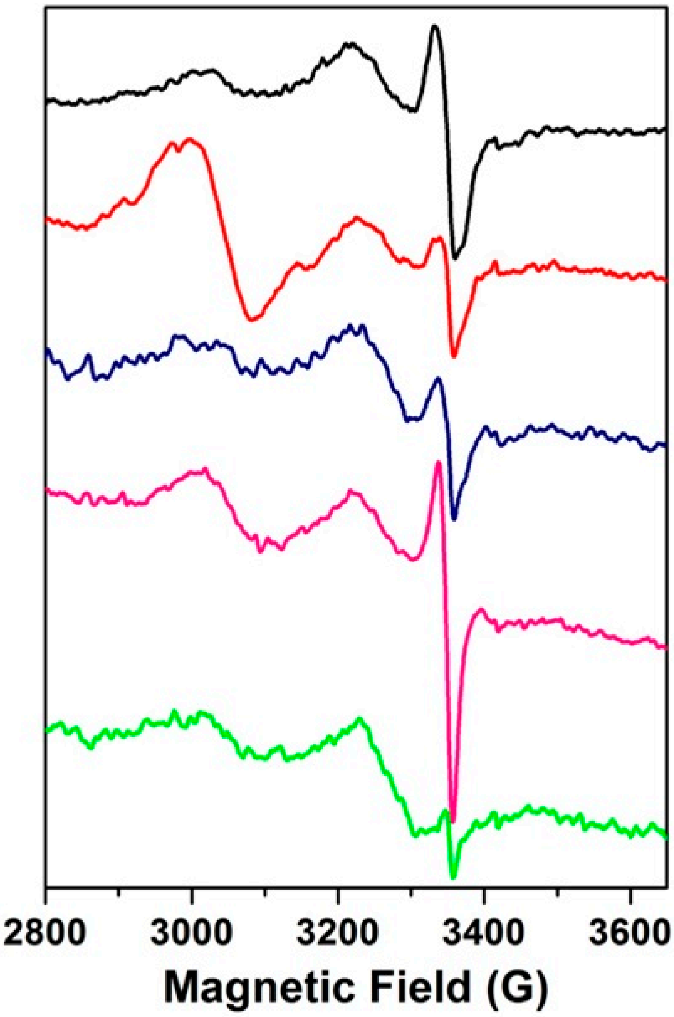

Figure S4. X-band EPR spectra of F598H (black), S594H (red), S594H-GP (blue), EFG-F598H (pink) and YGP-F598H (green) at 2 K in the presence of ~1 equiv. of KO_2 (microwave frequency, 9.49 GHz; microwave power, 20mW; modulation amplitude, 5 G).

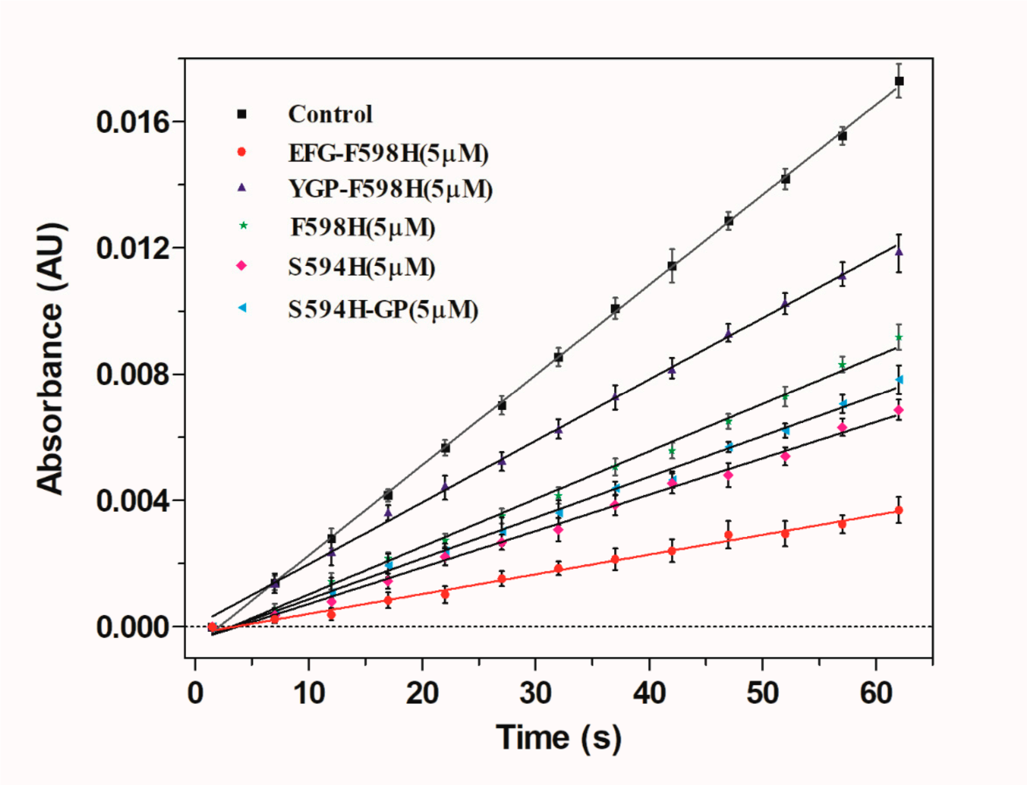

Figure S5. Measurement for SOD activity are using xanthine oxidase/NBT system. Black square: control, red circle: EFG-F598H, blue triangle: YGP-F598H, green star: F598H, magenta square: S594H, cyan triangle: S594H-GP. The results were presented as means ± SEM.

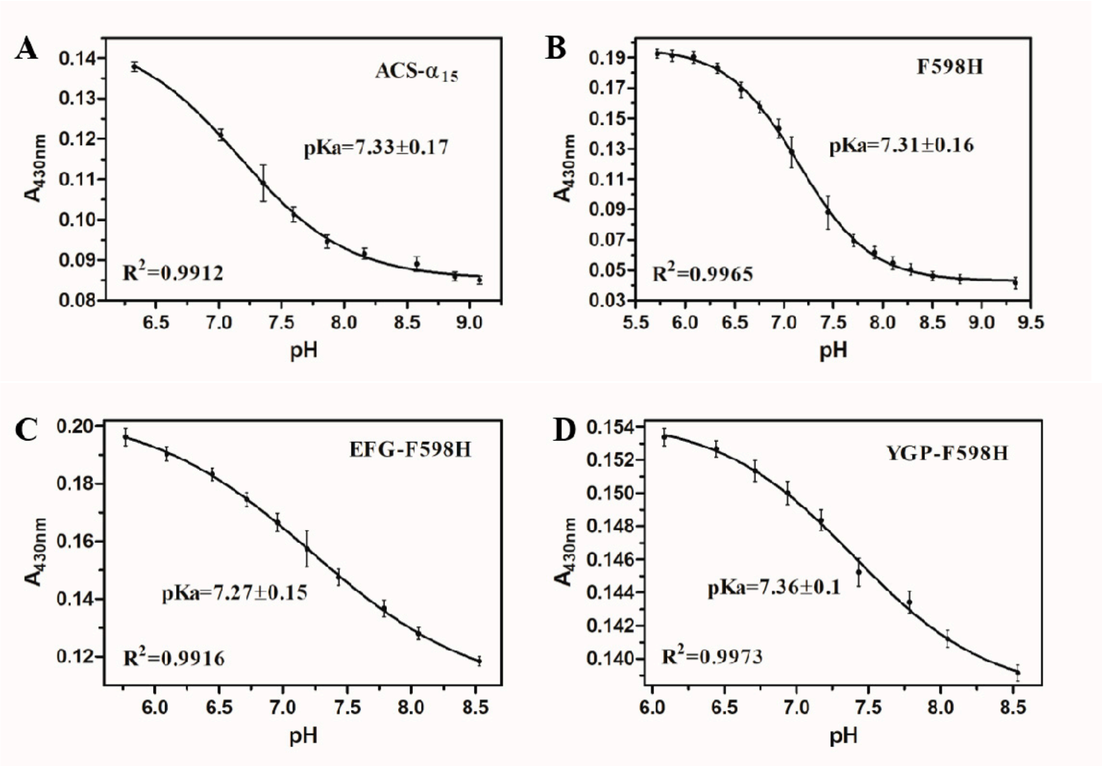

Figure S6. Optical pH titration based on the change in absorbance at 430 nm of ACS-α_{15}, F598H, EFG-F598H, and YGP-F598H. The results were presented as means ± SEM.

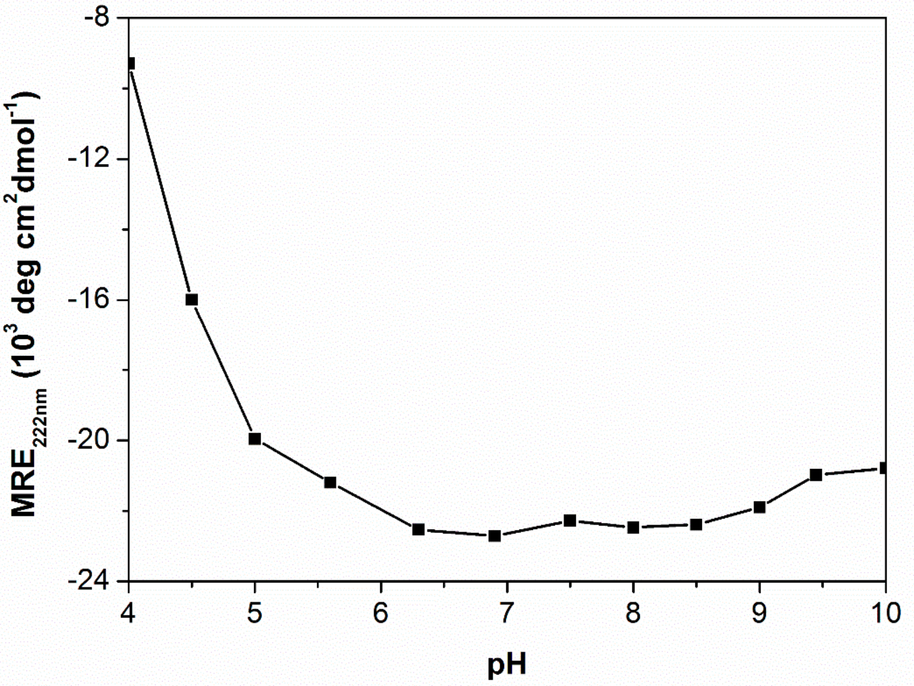

Figure S7. pH dependent changes of EFG-F598H in mean residual ellipticity (MRE) at 222 nm.

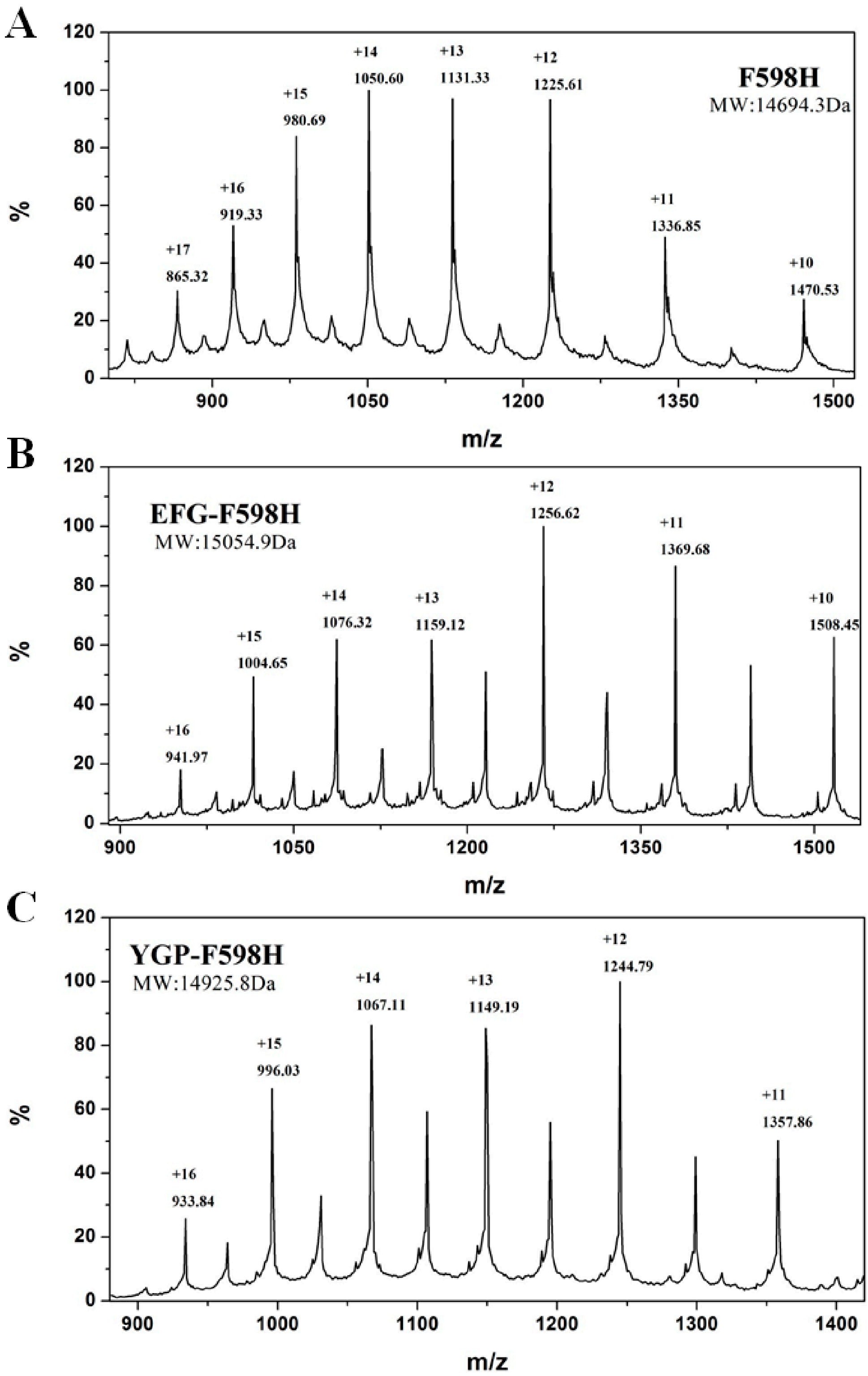

Figure S8. ESI-MS (positive ion mode) of solutions of F598H (A), EFG-F598H (B) and YGP-F598H (C) purified under aerobic conditions.

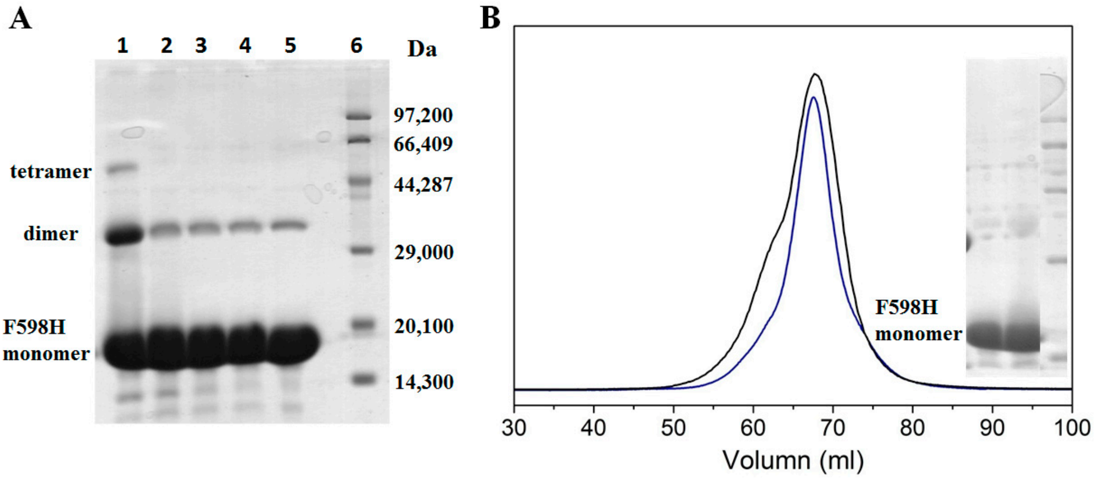

Figure S9. A. Non-reduced SDS-PAGE results of F598H purified under aerobic condition without reductant (lane 1), lane 2-5 show the results after TCEP added as a reducant with concentrations of 2, 10, 20, 50 mM. B. Gel filtration chromatography SuperdexTM 75 (HiLoad 10/160) of F598H purifed under aerobic condiation without reductant (black) and with reductant (blue). The insert show the non-reduced SDS-PAGE results of F598H purified with reductant.

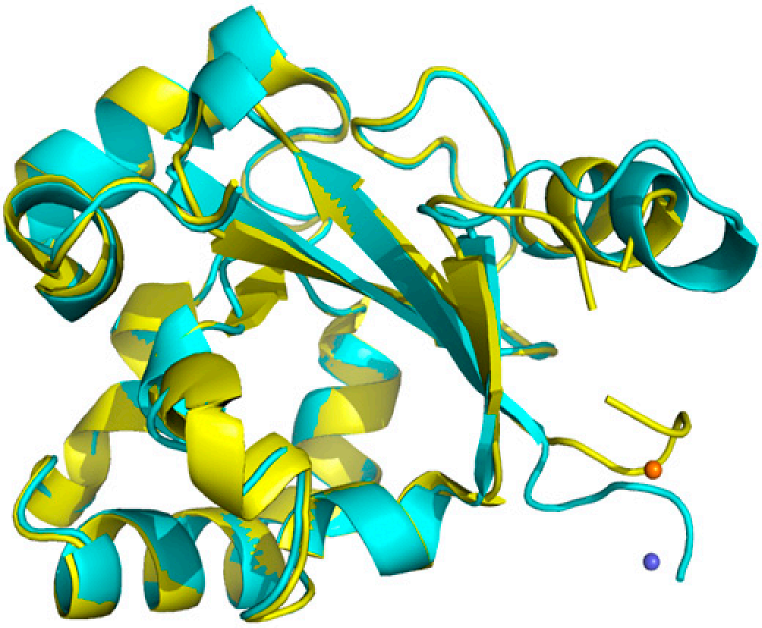

Figure S10. Superposition of F598H (cyan) and α_{15} (yellow).

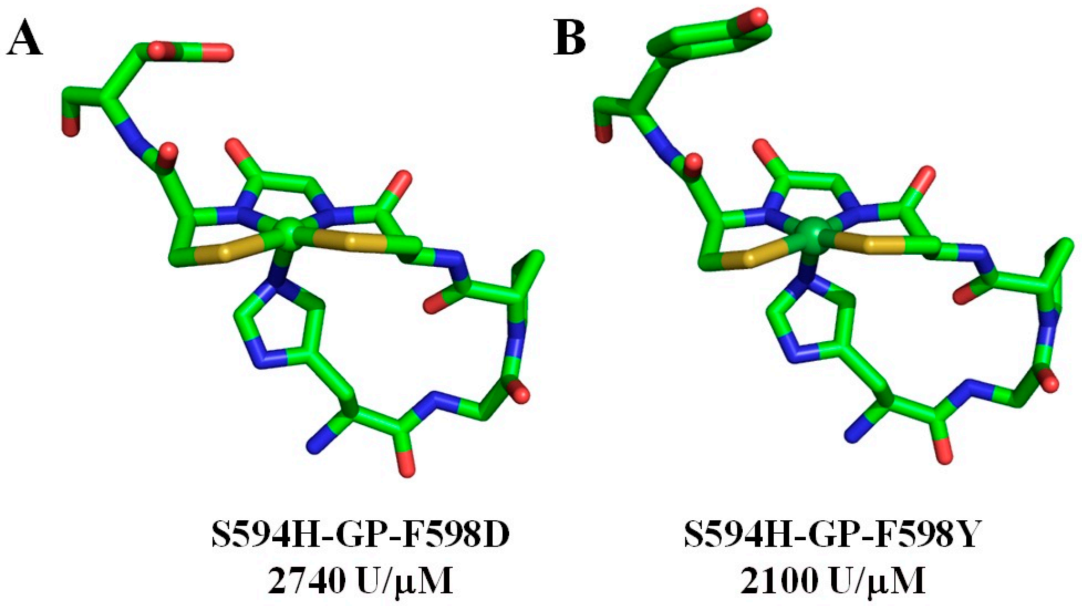

Figure S11. Structures of the molecular model of (A) S594H-GP-F598D and (B) S594H-GP-F598Y.

Table S1. PCR Primers.

	PCR Primers
P1	F: 5′-CGCCATATGATGGCTAGCATGTCGGACTCAG-3′<br>R: 5′-GCCTCATGGCAACCGCAGCTACCACCAATCTGTTCTCTGT-3′
P2	F: 5′-ACAGAGAACAGATTGGTGGTAGCTGCGGTTGCCATGAGGC-3′<br>R: 5′-CCGCTCGAGTCACATAATGGGATCCATGG-3′
P3	F:5′-GAATTCGGATGCGGTTGCCATGAGGCCATTATGG-3′<br>R:5′-ACTTCCACCACCAATCTGTTCTCTG-3′
P4	F:5′-TACGGACCTTGCGGTTGCCATGAGGCCATTATGG-3′<br>R:5′-ACCACCAATCTGTTCTCTGTGAGCC-3′
P5	F: 5′-TTCGAGGCCATTATGGCCATCCTGC-3′<br>R: 5′-GCAACCGCAGGAACCACCAATCTGT-3′
P6	F: 5′-CACTGCGGTTGCTTCGAGGCCATTA-3′<br>R: 5′-ACCACCAATCTGTTCTCTGTGAGCC-3′
P7	F: 5′-CACGGACCATGCGGTTGCTTCGAGG-3′<br>R: 5′-ACCACCAATCTGTTCTCTGTGAGCC-3′

Table S2. Data Collection and Refinement Statistics.

Protein name	F598H mutant PDB: 5GOL
Wavelength	1.486 Å Ni peak
Resolution range	31.24 - 2.11 (2.185 - 2.11)
Space group	C1 2 1
Unit cell(a, b, c;α,β， γ)	86.839, 58.569, 120.729; 90, 102.83, 90
Number of molecules per asymmetric unit Z	4
Unique reflections	36472
Multiplicity	8.4 (7.9)
Completeness (%)	98.59 (98.74)
Mean I/sigma(I)	20.1 (4.5)
Wilson B-factor	31.43
R-merge	0.124 (0.896)
R-pim	0.045 (0.327)
Reflections used in refinement	33783 (3366)
Reflections used for R-free	1981 (198)
R-work	0.2029 (0.2514)
R-free	0.2370 (0.2844)
Number of non-hydrogen atoms	4396
macromolecules	4069
ligands	24
solvent	303
Protein residues	540
total number of observations	294561
RMS(bonds)	0.016
RMS(angles)	1.67
Ramachandran favored (%)	97.74
Ramachandran allowed (%)	1.50
Ramachandran outliers (%)	0.75
Rotamer outliers (%)	3.17
Clash score	5.53
Average B-factor	34.56
macromolecules	34.28
ligands	42.76
solvent	37.57

Statistics for the highest-resolution shell are shown in parentheses.
